# BLAME-LESS STUDY: a two-arm randomized controlled trial evaluating the effects of an online psychoeducation programme for adolescents who have experienced physical/sexual violence or sexual abuse. Rationale, study design, and methods

**DOI:** 10.1080/20008066.2024.2315794

**Published:** 2024-02-19

**Authors:** Rik Knipschild, Helen Klip, Katie Winkelhorst, Tessa Stutterheim, Agnes van Minnen

**Affiliations:** a Karakter, Child and Adolescent Psychiatry, Almelo, the Netherlands; b Psychotrauma Expertise Centre (PSYTREC), Bilthoven, the Netherlands; c Behavioural Science Institute (BSI), Radboud University, Nijmegen, the Netherlands

**Keywords:** Trauma, shame, guilt, defense response, defense cascade, PTSD, psychoeducation, tonic immobility, appeasement, Trauma, vergüenza, culpa, respuesta de defensa, cascada de defensa, trastorno de estrés postraumático, psicoeducación, inmovilidad tónica, apaciguamiento

## Abstract

**Background:** Victims of physical/sexual violence or sexual abuse commonly experience defense responses that result in feelings of guilt and shame. Although trauma-focused interventions are effective in treating post-traumatic stress disorder symptoms, the presence of trauma-related shame and guilt can potentially hinder the process of disclosure during treatment, thus diminishing their overall effectiveness. It is hypothesized that providing psychoeducation about common defense responses will reduce feelings of shame and guilt, thereby increasing receptivity to trauma-focused treatment.

**Objective:** This paper describes the rationale, study design, and methods of the BLAME-LESS study. The effects of a brief online psychoeducation program will be compared with a waiting-list control group. The intervention aims to reduce feelings of trauma-related shame and guilt that adolescents experience regarding their own defense responses during and after physical/sexual violence or sexual abuse.

**Methods:** Adolescents (12 – 18 years old) with a history of physical/sexual violence or sexual abuse who suffer from trauma-related feelings of shame and guilt can participate in the study. The study follows a two-arm RCT that includes 34 participants. The primary outcomes includes trauma-related feelings of shame and guilt. The secondary outcomes includes PTSD symptoms, anxiety and depression symptoms, traumatic cognitions, readiness to disclose details of memories of the trauma, and motivation to engage in trauma-focused therapy. Assessments take place after screening, at baseline, two weeks after allocation to the intervention or waiting-list, and, only for the waiting-list participants, seven weeks after allocation to the intervention.

**Conclusions:** There is a need for treatment approaches that target trauma-related feelings of shame and guilt. A recently developed brief online psychoeducation program on defense responses during and after trauma offers victims of physical/sexual violence or sexual abuse a free and accessible way to obtain reliable and valid information. The proposed RCT will evaluate the effectiveness of this online psychoeducation program.

**Trial Registration:** Request is pending.

## HIGHLIGHTS

- Some defense responses to physical/sexual violence or sexual abuse, such as tonic immobility and appeasement behaviour, are common but unknown and raise feelings of shame and guilt.
- BLAME-LESS (In Dutch: On(t)schuldig) is a newly developed online psychoeducation programme that aims to reduce feelings of trauma-related shame and guilt. This programme includes explanatory animations, in-depth interviews with experts and victims, and written information accompanied by case reports.
- The proposed study examines the effectiveness of the brief online psychoeducation programme BLAME-LESS in a well-controlled study.

## Abstract (es)

**Antecedentes:** Las víctimas de violencia física/sexual o abuso sexual comúnmente experimentan respuestas de defensa que resultan en sentimientos de culpa y vergüenza. Aunque las intervenciones centradas en el trauma son eficaces para tratar los síntomas del trastorno de estrés postraumático, la presencia de vergüenza y culpa relacionadas con el trauma puede potencialmente obstaculizar el proceso de revelación durante el tratamiento, disminuyendo así su eficacia general. Se plantea la hipótesis de que proporcionar psicoeducación sobre las respuestas de defensa comunes reducirá los sentimientos de vergüenza y culpa, aumentando así la receptividad al tratamiento centrado en el trauma.

**Objetivo:** Este artículo describe la justificación, el diseño del estudio y los métodos del estudio MENOS CULPA (BLAME-LESS), un estudio controlado aleatorizado (ECA) de dos brazos. Los efectos de un breve programa de psicoeducación en línea se compararán con un grupo de control en lista de espera. La intervención tiene como objetivo reducir los sentimientos de vergüenza y culpa relacionados con el trauma que los adolescentes experimentan con respecto a sus propias respuestas de defensa durante y después de violencia física/sexual o abuso sexual.

**Métodos:** Se inscribirán en el estudio adolescentes (de 12 a 18 años) con antecedentes de violencia física/sexual o abuso sexual que sufran sentimientos de vergüenza y culpa relacionados con el trauma. El estudio sigue un ECA de dos brazos que incluye 34 participantes. Los resultados primarios incluyen sentimientos de vergüenza y culpa relacionados con el trauma. Los resultados secundarios incluyen síntomas de trastorno de estrés postraumático, síntomas de ansiedad y depresión, cogniciones traumáticas, disposición para revelar detalles de los recuerdos del trauma y motivación para participar en una terapia centrada en el trauma. Las evaluaciones se llevan a cabo después de la selección, al inicio, dos semanas después de la asignación a la intervención o lista de espera y, solo para los participantes en la lista de espera, siete semanas después de la asignación a la intervención.

**Conclusiones:** Existe la necesidad de enfoques de tratamiento dirigidos a los sentimientos de vergüenza y culpa relacionados con el trauma. Un breve programa de psicoeducación en línea desarrollado recientemente sobre respuestas de defensa durante y después del trauma ofrece a las víctimas de violencia física/sexual o abuso sexual una forma gratuita y accesible de obtener información confiable y válida.

## 1. Background

A meta-analysis (Alisic et al., 2014) indicate that 16% of children exposed to trauma develop post-traumatic stress disorder (PTSD). Among the different types of trauma, children who experience interpersonal trauma are at a higher risk of developing PTSD symptoms (Alisic et al., 2014; Nöthling et al., 2017). Interpersonal trauma refers to the experience of harm or distress within the context of a relationship or interaction with others, typically stemming from deliberate actions such as abuse or violence. Interpersonal trauma can have profound and lasting effects on an individual’s mental, emotional, and physical well-being. Importantly, individuals who have experienced interpersonal trauma often exhibit feelings of trauma-related shame and guilt (Holliday et al., 2018; MacGinley et al., 2019).

The experience of emotions, such as trauma-related shame and guilt, is often rooted in a child’s reactions during or after a traumatic event (Ehlers et al., 2003; Kernan & Sullins, 2022). These reactions can encompass feelings of self-blame, inadequacy, and a sense of responsibility for the occurrence of trauma. Individuals may internalize negative beliefs about themselves, attributing the traumatic event to their actions or perceived shortcomings. These reactions can be influenced by negative self-evaluations based on personal beliefs or societal expectations (Gilbert, 2019). These negative reactions might be explained by the Just World Theory (Lerner, 1980), in which people tend to see the world as fair, believing that good actions lead to positive outcomes and bad actions to negatives. This belief offers predictability and control, and individuals, including family and friends, may attribute blame to victims for maintaining their own beliefs. For instance, cases of sexual trauma commonly involve victim blaming, whereby the victim is held accountable for the outcome (van der Bruggen & Grubb, 2014). Victim blaming can manifest in various ways, such as attributing blame to the victim based on their clothing, suggesting that they contributed to the abuse, asserting that they did not explicitly say ‘no’, or failing to establish other clear boundaries. Consequently, victims may also engage in self-blame, assuming responsibility for actions that may have contributed to interpersonal trauma. However, it is worth noting that victim blaming tends to be more prevalent when victims do not actively defend themselves (e.g. fight) (Gravelin et al., 2018), which is often the case for interpersonal trauma.

In life-threatening situations, defense responses, such as fight or flight, encompass survival-based reflexive and automatic behavioural and physical reactions. In addition to the commonly known ‘fight-flight’ responses, individuals may exhibit several other defense responses during traumatic events. For instance, victims of physical/sexual violence or sexual abuse often experience a state of ‘freezing’, also known as ‘tonic immobility’ (TI) (Kalaf et al., 2017). Research has shown that victims frequently report TI as a perceived defense response during instances of sexual or physical violence (Hagenaars, 2016; Möller et al., 2017), and its presence during interpersonal trauma is linked to the severity of PTSD symptoms following trauma (Coimbra et al., 2023). Furthermore, according to Bracha (2004), the ‘fright’ and ‘faint’ responses are also considered defense responses. Kozlowska et al. (2015) identified various common defense responses as part of the defense cascade, including arousal, freezing, fight or flight, tonic immobility, collapsed immobility (threat-induced fainting), and quiescent immobility (a state of quiescence that promotes rest and healing). A recent literature review by Van Minnen (2017) addressed several defense responses specific to (repeated) interpersonal trauma, such as arousal, freezing, flight, fight, appeasement behaviour (including reconciliation and non-disclosure), and tonic immobility. These more unknown defense responses can be confusing, particularly in younger victims, and may lead to self-attributing questions. This is particularly the case regarding appeasement behaviour (questions like ‘Why did I go back to the perpetrator?’ ‘Why did I continue being nice to him?’ ‘Why did I not tell anyone?’) and tonic immobility (e.g. ‘Why did I not scream or fight?’).

The concept of defense cascade, a continuum of innate, hard-wired, automatically activated defense behaviours, and related defense reactions, is not well known among trauma victims or their social environment (Kozlowska et al., 2015). Lack of knowledge and misunderstanding of one’s defense responses can result in negative evaluations of these responses (Gilbert, 2019) and severe self-blame, as expressed by increased feelings of shame, guilt, dysfunctional post-traumatic cognition, and other PTSD-related symptoms. To illustrate, children and adolescents (5–16 years) who have been victims of interpersonal violence and who have feelings of guilt about whether or not they acted ‘right’ during the traumatic event experience more PTSD symptoms (Kletter et al., 2009). Moreover, negative cognitions and emotions, such as self-blame, shame, and guilt, can hinder the effectiveness of trauma-focused treatment (Øktedalen et al., 2015). Individuals who experience these feelings may avoid engaging in trauma-focused therapy altogether or withhold full disclosure of their reactions during and after a traumatic event. This is particularly evident among individuals aged 12–17 years, as they are less likely to disclose instances of sexual abuse than older individuals (Bicanic et al., 2015). While interventions targeting posttraumatic stress symptoms in children and adolescents have shown efficacy (John-Baptiste Bastien et al., 2020; Thielemann et al., 2022), trauma-focused interventions appear to be less effective when individuals experience pretreatment shame and guilt related to trauma (Øktedalen et al., 2015). This finding suggests that shame and guilt may impede recovery from trauma-related health problems. Notably, studies have demonstrated that changes in posttraumatic cognitions predict changes in posttraumatic stress symptoms (Schumm et al., 2015), and successful PTSD treatment is partly mediated by a reduction in the severity of trauma-related cognitions (Brown et al., 2018). Thus, it is crucial to implement specific interventions that address trauma-related shame and guilt prior to initiating trauma treatment, with psychoeducation being one such intervention that can encourage participation in therapy and facilitate disclosure, ultimately leading to improved outcomes.

Psychoeducation has been identified as a commonly used technique in the treatment of trauma-related symptoms (Kooij et al., 2022). Increased knowledge of reactions during sexual abuse has been associated with reduced victim blaming in adults, as measured post-intervention (Grandgenett et al., 2022). Additionally, a recently developed psychoeducation programme aimed at increasing knowledge about sexual and domestic violence, including common reactions to sexual abuse, resulted in fewer PTSD symptoms among adults immediately after the intervention (Laun, 2015). Similarly, in a controlled study involving adolescent victims of bullying, a short psychoeducation programme (4–6 h) led to immediate pre–post effects in decreasing self-blame and increased disclosure (Boulton & Boulton, 2017). Building on these findings, we hypothesized that providing psychoeducation about common defense responses as part of the defense cascade could contribute to a reduction in feelings of shame and guilt. Notably, to our knowledge, there are currently no standalone psychoeducation programmes specifically targeting the reduction of trauma-related shame and guilt related to one’s own defense responses during and after physical/sexual violence or sexual abuse. Consequently, we developed a brief online psychoeducation programme called BLAME-LESS (in Dutch: On(t)schuldig) (Stutterheim et al., 2022) that aims to educate adolescents about common defense reactions specific to (repeated) physical/sexual violence or sexual abuse, including arousal, freezing, flight, fight, appeasement behaviour (including reconciliation and non-disclosure), and tonic immobility (see Table 1 for a more detailed overview of the addressed defense reactions). We included podcasts, written information, and explanatory animations. Online interventions for trauma-related problems, as well as mental health problems in general, have become increasingly common and effective (Zhou et al., 2021), further enhancing the accessibility of psychoeducation programmes.

**Table 1.** Overview of the intervention.

Module	Main components
Module 1:	**Written information**
	*Content*
	Written information on the webpage explaining several defense responses that can occur during and after physical/sexual violence or sexual abuse; (1) during: arousal, freezing, flight, fight, appeasement behaviour (including reconciliation and secrecy), and tonic immobility. (2) after: attentional threat bias, vigilance, avoidance, hostility and embitterment, revictimization, shame, blame and nondisclosure, and freeze, numbing and amnesia. The written information is accompanied by illustrative case reports
	*Effort*
	The expectation is that module 1 will require approximately 60 min of time investment by the participant
Module 2:	**Animations**
	*Content*
	One visualized animation with voice-over about the defense cascade
	Two in-depth explanatory animations with voice-over of the most common defense responses: freeze/tonic immobility and appeasement behaviour
	*Effort*
	The expectation is that module 2 will require approximately 15 min of time investment by the participant
Module 3:	**Podcasts**
	*Content*
	Two in-depth interviews with trauma-experts about the defense cascade and how defense responses are unintentional, involuntary, and automatic responses with the purposes to survive life threating situations
	Three in-depth interviews about the defense cascade with two female adolescent survivors (age 18 and 20) of violence and sexual abuse
	*Effort*
	The expectation is that module 2 will require approximately 30 min of time investment by the participant

The primary objective of the BLAME-LESS study is to examine the effectiveness of an online psychoeducation programme, BLAME-LESS, compared to a waiting-list control group of adolescents (12–18 years), who experienced physical/sexual violence or sexual abuse, in reducing trauma-related feelings of shame and guilt. The second objective of the research is to examine the effects of the intervention on PTSD symptoms, anxiety and depression symptoms, and post-traumatic cognitions, including self-blame. Finally, by learning about common defense responses, we hypothesized that the online psychoeducational programme would increase adolescents’ willingness to disclose their trauma history and enhance their readiness and motivation to participate in trauma-focused treatment.

## 2. Methods

### 2.1. Study design

**Figure 1.**
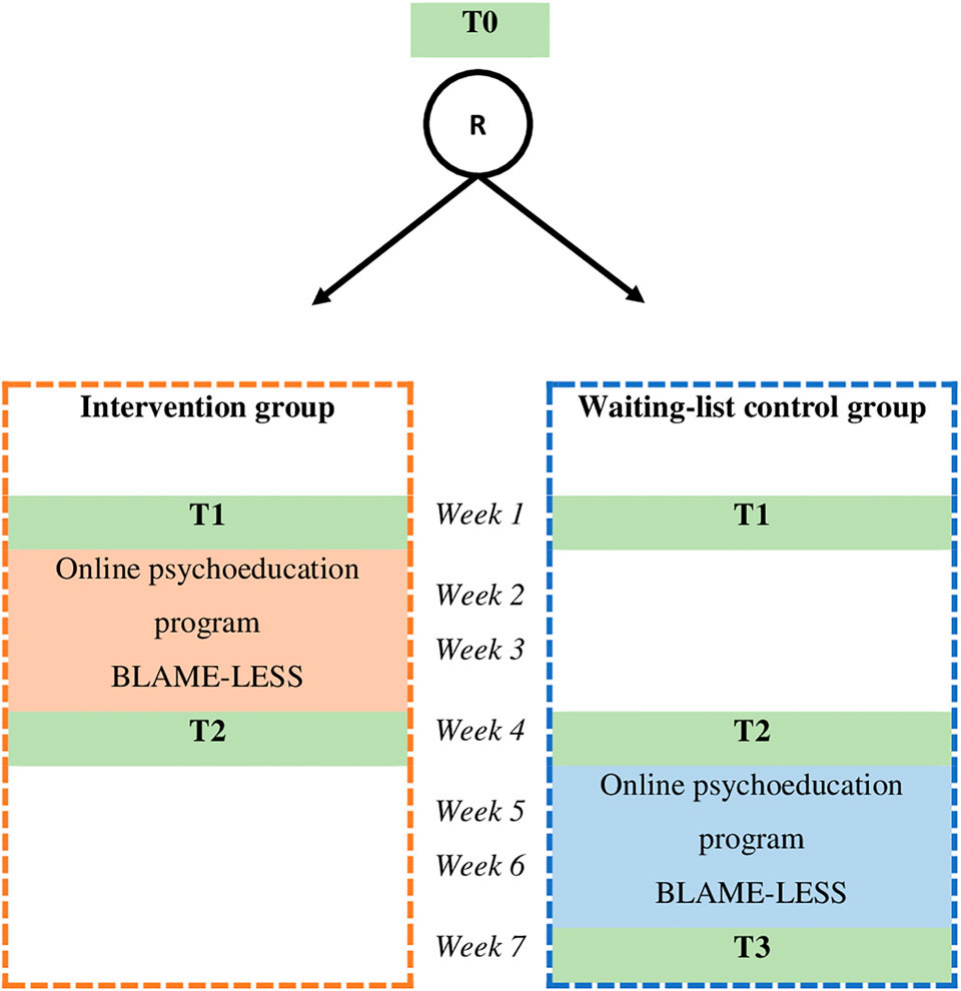
Study design.

An RCT will be conducted to study the effectiveness of a brief online psychoeducation programme called BLAME-LESS. The psychoeducation programme is an eHealth module with no active therapist involvement. Participants will be randomized into either the intervention group or the waiting-list control group. If allocated to the intervention group, participants will have direct access to the online intervention for two weeks after allocation. When a participant is assigned to the waiting-list control group, he or she has a two-week waiting period, after which the participant will have access to the intervention. Assessments take place after screening (T0), at baseline (T1), and two weeks after allocation (T2). Participants in the waiting-list group will also be assessed two weeks after they have gained access to the intervention (T3). Figure 1 provides an overview of the study design. The study protocol has been approved by the Medical Ethical Committee Oost-Nederland in the Netherlands (NL82795.091.22).

### 2.2. Research setting

The study was funded by Fonds Slachtofferhulp (grant number ‘20.08.18 deel 2’) and will be coordinated and executed at the Karakter Academic Centre for Child and Adolescent Psychiatry in the Netherlands, which includes 13 outpatient departments in four regions of the Netherlands.

### 2.3. Participants

Participants are adolescents between the ages of 12 and 18 who are referred to one of the outpatient departments. Participants may be referred by a general practitioner, pediatrician, or another youth care professional. Reasons for referral can vary, ranging from specific referrals for the current research project to general referrals for diagnostic psychiatric assessment and/or treatment. In all cases, participants and both parents (or legal guardians) provided consent for referral.

All participants meet the following criteria: (1) the participant experienced one or more traumatic life events, including physical/sexual violence or sexual abuse as indicated by the CTSQ; (2) the participant reported one or more trauma related feelings of guilt and/or shame on the CERQ (subscale ‘blaming oneself’: items 1 and/or 10 and/or 19 and/or 28), and these feelings are related to their defensive responses as indicated by a score of >4 on the VAS-scale; (3) the participant being fluent in written and spoken Dutch; (4) the participant is motivated and available for a period of seven weeks to engage in the study, including the psychoeducation programme BLAME-LESS, and commit to the assessments; and (5) The participant has the option to participate in the intervention on their own computer or smartphone. Exclusion criteria are: (1) acute suicidal behaviour or suicidal ideations requiring immediate hospitalization; (2) has already read the book that this programme is based upon: ‘Paralyzed with Fear’ (In Dutch: Verlamd van Angst by Agnes Van Minnen, 2017) or has been intensively informed otherwise in the past 12 months by a psychologist about common defense responses during and after trauma; (3) Cognitive Impairments (IQ < 70); (4) a brother or sister in the present study to avoid the potential transference of treatment effects (contamination bias) if siblings are randomized in different conditions (Magill et al., 2019); and (5) the adolescent is already involved in trauma-focused treatment.

### 2.4. Procedure

The first step of the procedure is to screen all adolescents referred to one of the outpatient departments. Part of the referral procedure is to complete an enrolment questionnaire. The enrolment questionnaire is a standard component of the clinical procedure that all parents (or legal guardians) and adolescents fill in, and this procedure is also used to check for inclusion and exclusion criteria for various research projects. The research assistant will check the following screening tools within the enrolment questionnaire: Child Trauma Screening Questionnaire (CTSQ), Cognitive Emotion Regulation Questionnaire (CERQ), and Visual Analog Scale (VAS). The research assistant will then check whether the inclusion and exclusion criteria are met. Part of the enrolment questionnaire is also that both the adolescent and the parents or legal guardians (for adolescents between the age of 12–16) can indicate if they give permission to be contacted by the researcher. If the adolescent (or the parents/legal guardians) declined, the data obtained from the enrolment questionnaire are solely utilized by the intake professional at the commencement of the treatment process.

When the screening data indicate that an adolescent qualifies for the study, and the adolescent and both parents or legal guardians agree (only necessary for adolescents age 12–16) to be approached for research purposes, contact will be initiated by a research assistant to discuss participation in the study. Throughout all instances, participants are explicitly apprised of the voluntary nature of completing the screening questionnaires for research purposes. Furthermore, the participant is informed in advance that if they are between 12 and 16 years old and consent to being contacted to discuss participation in scientific research, their parents or legal guardians will also be informed. Apart from being a legal requirement, this also aims to prevent participants from disclosing events that they may or may not want to inform their parents about. This minimizes the risk of parents being newly informed about their child’s victimization by the research team. It should also be acknowledged that this approach may potentially result in limited reach to a subset of adolescents, primarily because of constraints stemming from their current circumstances that prevent full disclosure. Conversely, individuals aged 16 years and older are governed by distinct legal and regulatory regulations. Within our jurisdiction, these participants hold the autonomy to independently determine their participation in scientific research. Commencing the procedural sequence, we ascertained their preferred mode of contact and we will not inform their parents or legal guardians.

When meeting the inclusion criteria, the participants will receive a letter with information about the intervention, study, and informed consent form. Parents (or legal guardians) from participants between the ages of 12 and 16 years will also receive a letter with information and will be asked for permission. Each participant can individually start with the intervention whenever they are ready. After inclusion and obtaining informed consent, participants will have one week to complete the baseline questionnaires (T1). Once they completed the baseline questionnaires, the randomization procedure will start. Participants will be randomly allocated to the intervention or waiting-list control groups. The Castor EDC will be used as a random-number generator. A 1:1 allocation ratio will be applied. We utilize block randomization with varying block sizes for this purpose, stratifying for the following variables: gender (boy, girl), type of trauma (physical violence, sexual abuse), and frequency of trauma (single occurrence, multiple occurrences) to ensure that the number of participants is well balanced across both treatment arms within each stratum. Randomization results will be communicated to the participants and their parents (or legal caretakers) via e-mail or phone calls. After the randomization procedure, participants will receive login codes on a website where they can engage in the online psychoeducation programme. The website is available through a login, which allows the research team to evaluate the parts of the website that have been visited and the time spent on the webpage. After two weeks, measurements will be taken (T2) for both groups. After T2, the participants referred to the waiting-list control group will receive access to the online psychoeducation programme. Two weeks after access, post-intervention measurements will be conducted (T3) for the waiting-list control group. Completing the questionnaires takes approximately 15–30 min per assessment. There are no costs incurred by the participants. Participants who dropout before the start of the intervention from the study will be replaced by new participants, with a maximum of 20% of all participants. Participants who dropout during the intervention will not be replaced.

### 2.5. Intervention BLAME-LESS

All participants will receive the online psychoeducation programme BLAME-LESS. This programme was developed in collaboration with trauma experts and adolescents who survived physical/sexual violence or sexual abuse. The programme was designed to educate about common defense responses that may occur during and after physical/sexual violence or sexual abuse. The main goal of the programme is to reduce the feelings of shame and/or guilt related to these responses. All aspects of the programme were created in collaboration with licensed developmental and clinical psychologists with extensive psycho-trauma expertise, adolescent experienced experts, visual artists, and web designers. The programme is offered via a website and consists of three modules (Table 1).

To ensure a comprehensive and effective learning experience, the BLAME-LESS programme integrates multimodal approaches. It incorporates a combination of different learning styles, including visual, auditory, and online reading activities. By catering to individuals with varying preferences, this approach provides a well-rounded learning experience that enhances engagement and knowledge retention.

The development of the BLAME-LESS programme also benefits from expert input. Guidance and information from professionals in relevant fields, such as psychologists, therapists, counsellors, and subject matter experts, are sought. Their expertise contributes to evidence-based knowledge, research findings, and best practices to ensure the programme’s effectiveness and credibility. In addition, personal testimonials play a crucial role in the BLAME-LESS programme. Real-life stories and experiences shared by adolescents who have experienced similar challenges or traumas are included. These personal narratives offer unique insights, relatability, and emotional connections for programme participants. By showcasing the journeys and perspectives of others who have faced similar situations, the programme fostered empathy, understanding, and a sense of community among its participants.

Some of the common elements addressed by Kooij et al. (2022) for evidence-based trauma therapy for children and adolescents are embedded in the online BLAME-LESS programme. Information about common defense responses and post-trauma reactions is provided (psychoeducation). In addition, the concept of cognitive shifting is used to influence or modify dysfunctional cognition by adding new information about the defense cascade.

The psychoeducational programme will take approximately 180 min to complete. Participants will receive a step-by-step guide on how to navigate the website and a checklist with all modules they can enrol in. It is advised that the participant will follow the psychoeducation programme while he/she is at home, and that they will have two weeks to watch, read, and listen to the entire programme on their own terms. They will be informed that the research team will track website use within the psychoeducation programme to monitor engagement. Participants will receive a login via email. The login code grants access to the website, which is provided in a restricted environment. Within this restricted environment, the research team ascertained which sections of the website had been viewed, and the duration the participant took for each section. The research team cannot track website use outside the psychoeducation programme environment. During the programme, a licensed psychologist can be contacted during working hours via e-mail and/or phone for their support and questioning. Contact information is provided through the information letter prior to the start of the study as part of the informed consent procedure. After the intervention, participants receive care as usual.

### 2.6. Measurements

Measures are administered at four time points: at screening (T0), at baseline (T1), two weeks after allocation to the intervention or waiting-list period (T2), and, only for the waiting-list participants, seven weeks after allocation to the intervention (T3). Questionnaires are distributed using Castor EDC and received by the participant via email.

An overview of the measurement instruments and time points is presented in Table 2.

**Table 2.** Measurement instruments and time points.

	Number of items	Maximum time to complete	Assessment time points
Screening			
Child Trauma Screening Questionnaire (CTSQ)	24 items	5 min	T0
Cognitive Emotion and Regulation Questionnaire (CERQ)	4 items	1 min	T0
Feelings of Shame and Guilt about Defensive Responses (VAS rating)	2 items	1 min	T0
Baseline			
Demographic information		NA	T1
Primary outcome			
Feelings of Shame and Guilt (SGATS)	9 items	5 min	T1, T2, T3*
Secondary outcomes			
Posttraumatic stress (KJTS)	25 items	10 min	T1, T2, T3*
Post-Traumatic Cognitions (CPTCI)	25 items	10 min	T1, T2, T3*
Motivations and Disclosure (TPEQ)	6 items	5 min	T1, T2, T3*
Anxiety and Depression (PROMIS)	16 items	5 min	T1, T2, T3*
Feelings of Shame and Guilt about Defensive Responses (VAS rating)	2 items	1 min	T1, T2, T3*
Other Questionnaires			
Feasibility Test	5 items	5 min	T2, T3**
Knowledge Test	6 items	1 min	T1/T2/T3*

Note. *Participants in the intervention group will not receive T3 measurements.

**For participants in the waitlist control group, this assessment will take place at T3.

#### 2.6.1. Screening for traumatic experiences (CTSQ)

The Child Trauma Screening Questionnaire (CTSQ; Kenardy et al., 2006) is a self-reported measure that contains a 14-item list of traumatic life events and a 10-item list to measure post-traumatic stress symptoms of re-experiencing and hyperarousal. Each life event can be answered with yes (scored as 1) or no (scored as 0). Scores >4 indicate positive screening for trauma symptoms. For screening, we will use only two items on the list of traumatic life events, namely items that refer to having experienced (sexual) violence and/or sexual assault (Items 1 and 2). The CTSQ has been shown to have good convergent validity (Kenardy et al., 2006). Internal consistency was reported, with a Cronbach’s alpha of .69.

#### 2.6.2. Cognitive Emotion Regulation Questionnaire (CERQ)

The Cognitive Emotion Regulation Questionnaire (CERQ; Garnefski et al., 2002) is a 36-item self-report questionnaire with five response categories: ‘(almost) never, sometimes, regularly, often, and (almost) always’. The items refer to what a person thinks when experiencing threatening or stressful events. The full questionnaire measured nine cognitive coping strategies. For screening, we will use only four items on the self-blame subscale (Items 1, 10, 19, and 28). Internal consistency of the self-blame subscale was alpha = .81 (high) for young adolescents (13–15 years) and .68 (medium) for older adolescents (16–18 years).

#### 2.6.3. Demographics

All children referred to child and adolescent psychiatry complete a comprehensive intake form and undergo an intake procedure. During this procedure, parents share demographic information to appropriately allocate care. In the present study, demographic information will be collected from medical files. The information will include age, sex, and school functioning.

#### 2.6.4. Shame and Guilt After Trauma Scale (SGATS)

The Shame and Guilt After Trauma Scale (SGATS; Aakvaag et al., 2016) measures trauma-related shame and guilt. The SGATS consists of nine items: four on trauma-related feelings of shame and five on trauma-related feelings of guilt (Aakvaag et al., 2016). Each question can be answered on a 3-point Likert scale (0 = no, 1 = yes, a little, 2 = yes, a lot). The minimum SGATS score is 0, and the maximum score is 18. The SGATS has a Cronbach’s alpha of 0.84 for feelings of shame and .87 for feelings of guilt. The SGATS was translated into Dutch using the five iterative stages described by Douglas and Craig (2007): translation, review, adjudication, pretesting, and documentation.

#### 2.6.5. Child and Adolescent Trauma Screening (KJTS-NL)

The Dutch version of the Child and Adolescent Trauma Screener (KJTS-NL; Kooij & Lindauer, 2019) is a questionnaire for children and adolescents that serves as a screening tool for PTSD symptoms (Sachser et al., 2017). The questionnaire consists of three parts. In the first part, adolescents are asked which major (traumatic) events they have experienced. In the second part, the severity of PTSD symptoms is measured with 20 items, rated on a 4-point Likert scale (range 1–4) and in the third part, the children and adolescents are asked to indicate (yes/no) whether the above-mentioned symptoms caused difficulties in certain daily activities. A total score below 15 suggests no (clinical) indication of the presence of a PTSD classification, a score between 15 and 20 suggests the possible presence of a clinical PTSD classification, and a score above 20 suggests an increased chance of the presence of a PTSD classification. Cronbach’s alpha for all components varies between .88 and .94 (Sachser et al., 2017). The results for the Dutch version of the KJTS are not yet available.

#### 2.6.6. Child Post-Traumatic Cognitions Inventory (CPTCI)

The Child Post-Traumatic Cognitions Inventory (CPTCI; Meiser-Stedman et al., 2009) is a self-report questionnaire that measures trauma-related cognition in children and adolescents. The questionnaire consists of two subscales (permanent and disturbing change subscale, CPTCI-PC; fragile person in a scary world subscale, CPTCI-SW) with a total of 25 items that can be answered on a four-point Likert scale, ranging from 1 (strongly disagree) to 4 (strongly agree). The English version of the CPTCI has been validated in children aged–6–18 years. The Dutch CPTCI has good reliability and validity (Diehle et al., 2015), high internal consistency (Cronbach’s alpha 0.86–0.93), and good convergent validity.

#### 2.6.7. Treatment Program and Evaluation Questionnaire (TPEQ)

The Treatment Program and Evaluation Questionnaire (TPEQ; Murphy et al., 2009) assesses readiness and motivation for trauma-focused treatment (Murphy et al., 2009). For the present study, we use four items (items 8 through 11 have been incorporated) from the TPEQ. Additionally, the two following items on Disclosure have been added to the questionnaire: ‘To what extent do you think: I will disclose everything during the treatment, even the things I feel ashamed of?’ and ‘To what extent do you think: I will disclose everything during the treatment, even the things I feel guilty about?’ All questions were answered on a 7-point Likert scale (ranging from 1 = strongly disagree to 7 = strongly agree). A higher score indicates greater willingness, motivation for treatment and readiness for disclosure in treatment.

#### 2.6.8. Patient-Reported Outcomes Measurement Information System

The Patient-Reported Outcomes Measurement Information System (PROMIS; Terwee et al., 2014) measures Anxiety and Depression symptoms. The lists consist of a dynamic set of item banks based on Computerized Adaptive Testing (CAT). Both questionnaires consist of 8 items. Each question can be answered as follows: never, almost never, sometimes, often, and very often. PROMIS is a short form of CAT, primarily utilized for research purposes. In the current study, the raw item scores will be summed, resulting in a PROMIS total score. Higher scores indicate more severe anxiety and/or depression symptoms. The calculated PROMIS total scores from different measurement points will be compared.

#### 2.6.9. Feelings of shame and guilt: visual analog scale

The participants rated guilt and shame on a by the authors developed 10-point visual analogue scale (VAS; 1 = no, not at all to 10 = yes, very much). Item 1: ‘Do you experience feelings of guilt regarding your own reactions during (or after) the stressful event, such as what you said and did, or perhaps what you did not say or do? Item 2: ‘Do you experience feelings of shame regarding your own reactions during (or after) the unpleasant event, such as what you said and did, or perhaps what you did not say or do?’

#### 2.6.10. Feasibility

We developed a short questionnaire with five questions that will be used to assess whether the BLAME-LESS programme intervention is attractive and easy for participants to use. We aim to assess whether the intervention was perceived as appealing (1) by the participants. We will explore how the participants experienced the tone of texts (2) and whether the animation resonated (3) with them. Furthermore, we will inquire whether the youth visited (4) all sections of the website and whether the information provided was clear (5). These open-ended questions are posed to the participant through an online form after the intervention’s completion. The information provided may be used to further adjust or optimize the intervention.

#### 2.6.11. Knowledge assessment

To ascertain the extent of knowledge enhancement pertaining to survival responses following the intervention, participants will be required to complete a brief knowledge questionnaire both prior (T1) to and subsequent to the intervention (T2 or T3). The internally devised questionnaire comprises six items structured on a 7-point Likert scale. Participants are afforded the opportunity to express their level of agreement or disagreement with statements, ranging from complete agreement to complete disagreement, or any point in between. Statements include assertions such as ‘Freezing or immobilization is an automatic response under stress that one cannot control’ and ‘During instances of physical/sexual violence or sexual abuse, automatic bodily reactions occur with the aim of survival, over which the individual possesses minimal control.

### 2.7. Data analyses

The G*Power programme (version 3.1.9.2) was used to calculate the sample size. An a priori power calculation showed that the proposed RCT sample required 34 participants (*n* = 17 for each condition). Calculations in G*Power (Cohen’s *d* = 0.5; equivalent of *f* = 0.25 ANOVA) showed that a sample size of *n* = 17 for each group had sufficient power (80%) to reject our null hypothesis, namely, that there was no difference between the two conditions. A between groups effect size of Cohen’s *d* of 0.50 is considered a medium effect size. In a comparable study (Boulton & Boulton, 2017), the between group pre–post effect size on self-blame was very large (*d* > 1.2) and the sample size was also comparable. Based on this study, we believe that the expected effect size (Cohen’s *d*) of .50 in our study is reasonable.

Data will be analyzed using SPSS V.24.0 for Windows (SPSS Incorporated). Descriptive statistics will be calculated for the baseline characteristics of the study population. Parametric data are presented as means with standard deviations (SD), and nonparametric distributed variables as medians with Inter Quartile Ranges (IQRs). The intervention effect will be evaluated for group differences between Condition 1 (intervention) and Condition 2 (waiting-list control group). The primary outcome measure is feelings of shame and guilt, as measured with the SGATS. We will use a will be tested using an two-way repeated measures ANOVA test to determine whether any change in response to the SGATS (i.e. the dependent variable) is the result of interaction between the ‘type of treatment’ (i.e. the waiting-list control group or Intervention group, which is one of our two factors) and ‘time’ (i.e. our second factor T1 and T2). Statistical significance was set at *P* < .05. The secondary outcome measures included the severity of posttraumatic cognition symptoms, posttraumatic stress symptoms, anxiety, mood symptoms, and changes in motivation for trauma-focused treatment and disclosure. Changes between the groups at pre-test and post-test will be tested using ANOVA, while correcting for multiple testing.

## 3. Discussion

The objective of the present study is to outline the rationale, study design, and methods employed in the BLAME-LESS study, a two-arm randomized controlled trial (RCT) assessing the impact of a concise online psychoeducation programme. This programme aims to alleviate feelings of trauma-induced shame and guilt experienced by adolescents concerning their defense responses during and following instances of physical/sexual violence or sexual abuse. These experiences of physical/sexual violence or sexual abuse are recognized as severe adverse events with potentially enduring effects on the development of a child (Downing et al., 2021; Hughes et al., 2017). Equipping adolescents with reliable information and valid explanations of typical defense responses that may arise during and after physical/sexual violence or sexual abuse is crucial for their journey to recovery. Our newly developed online psychoeducation programme, BLAME-LESS, affords young survivors of physical/sexual violence or sexual abuse with the opportunity to explore fresh perspectives on their reactions to traumatic experiences. By attaining knowledge about the defense cascade, we hypothesize that the dysfunctional posttraumatic cognitions and emotions associated with their trauma will diminish. To achieve this, the current programme incorporated several established components widely recognized in evidence-based psychological treatment of trauma-related stress in children and adolescents.

The proposed study has several strengths. First, it aims to evaluate the effectiveness of an online psychoeducation programme. A recent meta-analysis (Rogers et al., 2017) revealed a wide range of evidence-based internet programmes. However, most of these programmes are not easily accessible to the public (Rogers et al., 2017). If the proposed randomized controlled trial demonstrates a reduction in trauma-related shame and guilt, the online psychoeducation programme BLAME-LESS will become publicly available. This will offer a cost-effective and user-friendly solution by providing evidence-based online intervention that is accessible and free to use. Second, the study employed an RCT design with a waiting-list control group. By including a waiting-list control group, we can assess the effectiveness of the intervention using a well-controlled design. Furthermore, the BLAME-LESS programme has the advantage of requiring minimal time commitment. It incorporates various modules that cater to different learning styles, including visual, auditory, and online reading activities. This allows for a comprehensive learning experience. If effective, the programme can be implemented during potential waiting periods for treatment or as a precursor to psychological intervention.

While this study exhibits particular strengths, it is not exempt from certain limitations. A constraint arises from the online delivery of the intervention, lacking the direct involvement of a therapist. However, it is crucial to note that the specific aim of the intervention is for it to be independently viewed by adolescents, without the need for direct therapeutic guidance. Additionally, the study relies on self-reported measures, which can lead to potential overestimation or underestimation of symptoms. Future research should also incorporate parental input into outcome measures and examine how incorporating a support system might contribute to treatment outcomes. Furthermore, the limited sample size and absence of follow-up assessments restrict the generalizability of the findings. We did not include follow-up procedures due to ethical concerns regarding the potential burden on subjects relative to the study’s objectives. It is crucial for future research to explore the generalizability of the results, investigate long-term effects, and examine the impact on trauma-focused treatment when incorporating the BLAME-LESS programme prior to such treatment. Finally, no psychometric properties of the SGATS questionnaire are known for the target population of this study.

However, the current study is valuable for clinical practice and public health. The results will help evaluate the added value of accessible and free online psychoeducation for adolescents suffering from trauma-related guilt and shame. By identifying ways to reduce feelings of shame and guilt, adolescent survivors of physical/sexual violence or sexual abuse may be more willing to seek help and disclose important trauma-related information, leading to better treatment outcomes.
